# Supplementary material for: Precise Topology of Adjacent Domain-General and Sensory-Biased Regions in the Human Brain
Source: Cereb Cortex. 2021 Oct 9;32(12):2521–37. doi: 10.1093/cercor/bhab362 (PMC9201597; doi:10.1093/cercor/bhab362)
Supplement: Supplementary_figures_bhab362 [file supplementary_figures_bhab362.pdf]

Supplementary figures

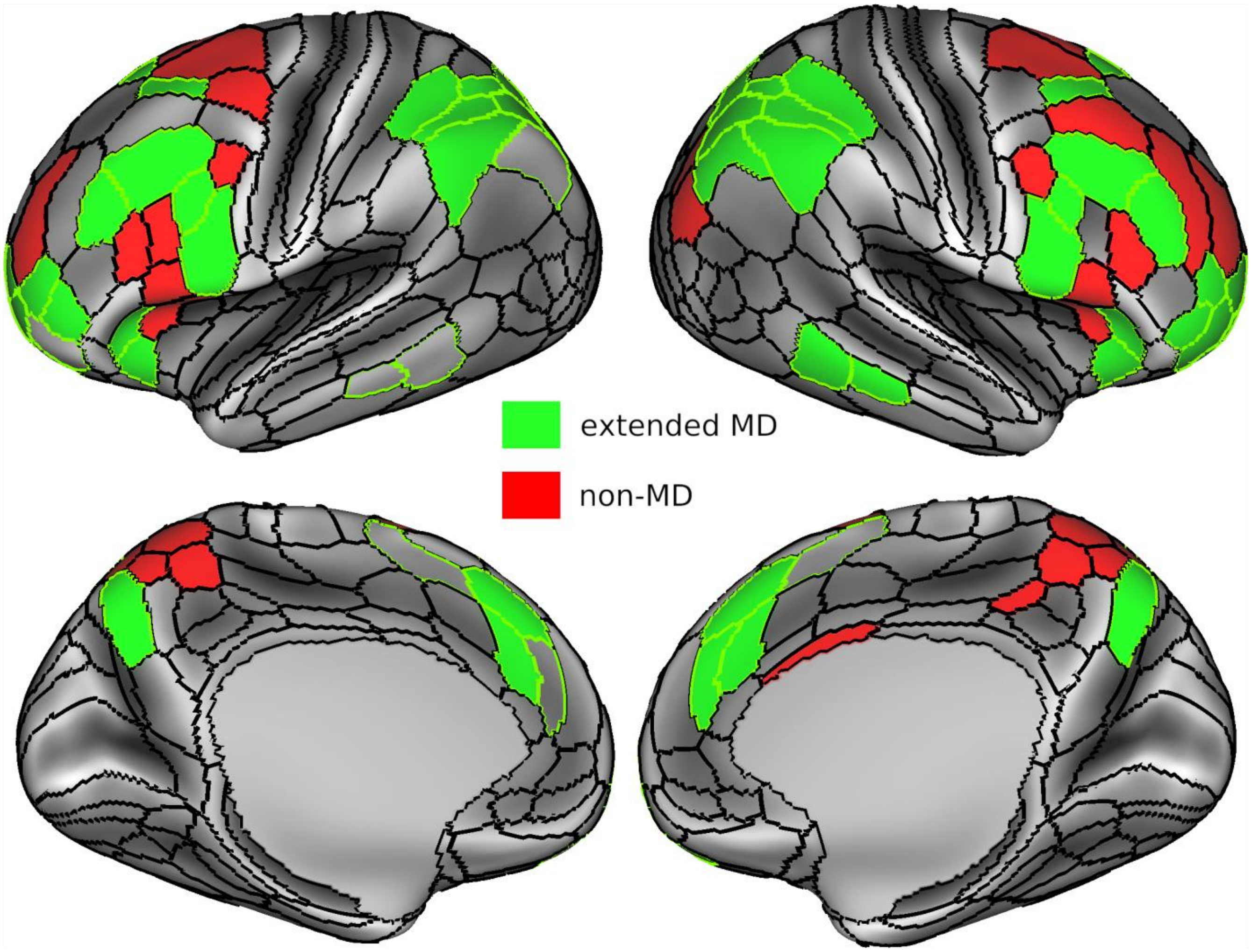

Supplementary figure 1. Conjunction of significantly activated parcels ($p<0.05$, Bonferroni corrected for 360 parcels) in both visual and auditory hard>easy contrast. Extended MD regions are in green, non MD regions are in red. Black contours belong to the borders of the HCP MMP1.0, green contours belong to borders of extended MD regions. Data is available at https://balsa.wustl.edu/G3l24

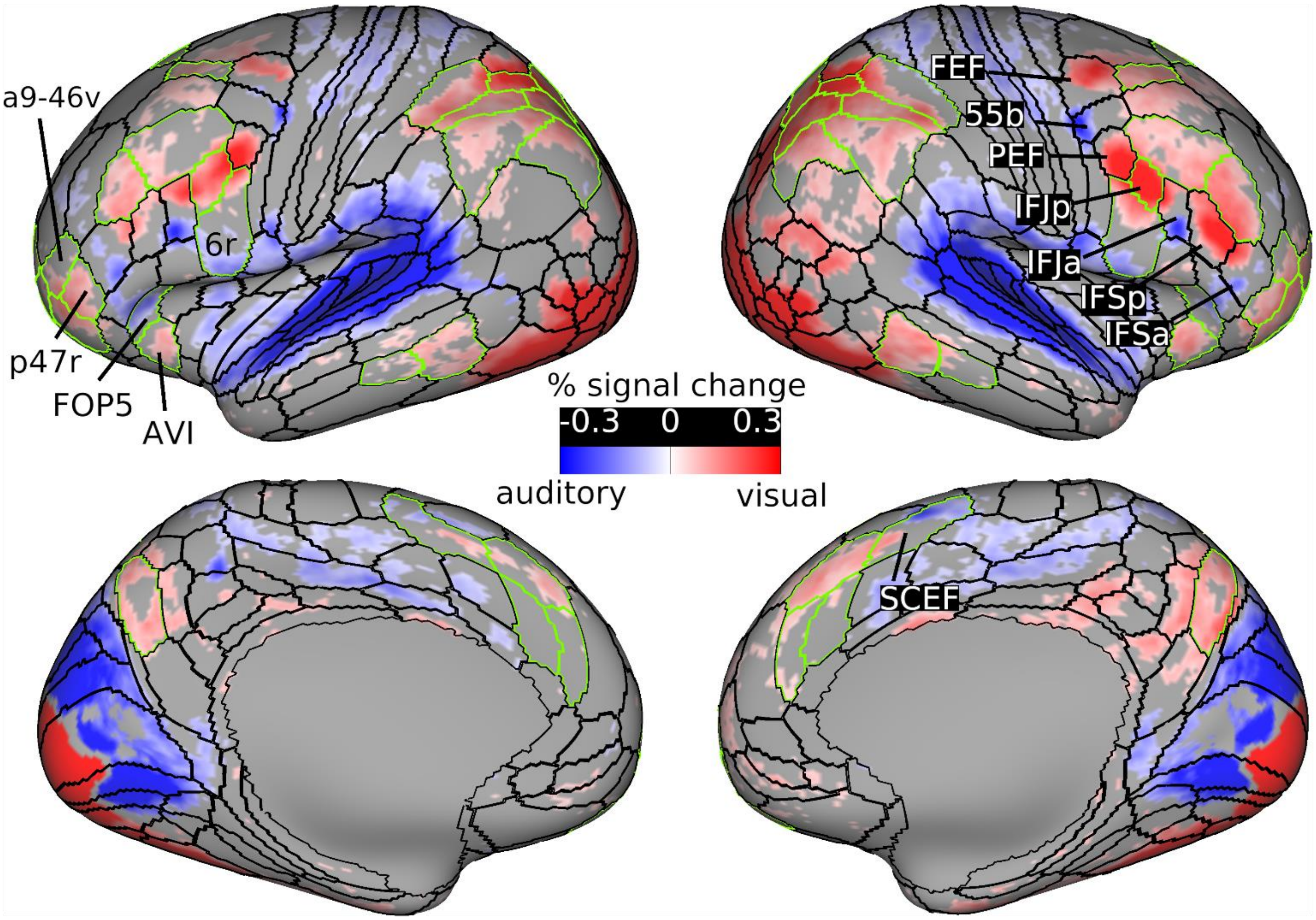

Supplementary figure 2. Significant vertices (colored) for the visual easy>fix minus auditory easy>fix contrast (FDR corrected p<0.05). Activations are % signal change. Black contours correspond to the HCP MMP 1.0 areal borders and green contours correspond to extended MD areal borders. Data is available at https://balsa.wustl.edu/zpj8K

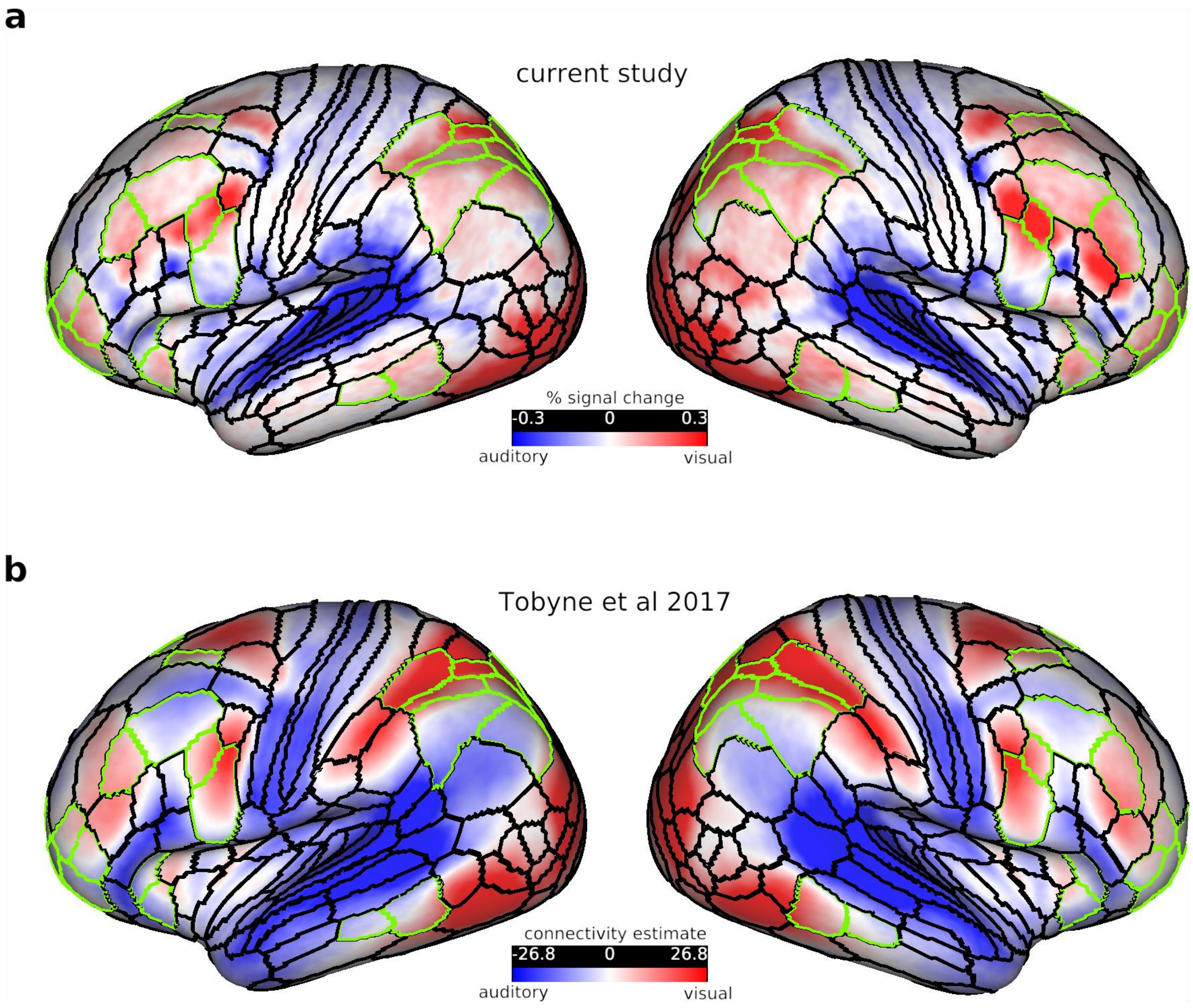

Supplementary figure 3. (a) easy>fix visual vs easy>fix auditory activations from current study. (b) Auditory vs visual modality biases as defined by Tobyne et al (2017) using rfMRI connectivity (see Tobyne et al 2017 for details on methods). Black contours correspond to the HCP MMP 1.0 areal borders and green contours correspond to extended MD areal borders. Data is available at https://balsa.wustl.edu/X5Pjj
